# Sphingosine-1-phosphate expression in human epiretinal membranes

**DOI:** 10.1371/journal.pone.0273674

**Published:** 2022-08-31

**Authors:** Minho Kim, Soonil Kwon, Sohee Jeon, Byung Ju Jung, Kyu Seop Kim

**Affiliations:** 1 Apgujung St. Mary’s Eye Center, Seoul, Republic of Korea; 2 Department of Ophthalmology, Hallym University Sacred Heart Hospital, Anyang, Republic of Korea; 3 Keye Eye Center, Seoul, Korea; Tsukazaki Hospital, JAPAN

## Abstract

The abnormal posterior vitreous detachment (PVD) is speculated as an important mechanism of the development of the epiretinal membrane (ERM). However, there is only limited information about the molecular mechanism. Sphingosine-1-phosphate (S1P) is a mediator of the mechanosensitive response in several cell types that may have a role in the pathogenesis of ERM during abnormal PVD. Therefore, we evaluated the expression of S1P in the human ERM and the role of S1P in cultured human Muller glial cells. Among 24 ERM specimens, seven specimens (29.2%) exhibited S1P expression. Patients with secondary ERM or ellipsoid zone defects, which suggest abnormal PVD presented a significantly higher S1P+ cell density (secondary ERM: 128.20 ± 135.61 and 9.68 ± 36.01 cells, *p* = 0.002; EZ defects: 87.56 ± 117.79 vs 2.80 ± 8.85, *p* = 0.036). The addition of S1P increased the migrative ability and expression of N-cadherin and α-SMA in human Muller glial cells, suggesting S1P is a potential causative molecule for the development of ERM during abnormal PVD.

## 1. Introduction

Epiretinal membrane (ERM) is a common condition of cellular proliferation and extracellular matrix deposition on the inner retinal surface [1–3]. Although the exact pathogenesis is not fully understood, most demographic studies have agreed that age, posterior vitreous detachment (PVD), and cataract surgery are key factors that influence ERM prevalence [1–3]. As retinal imaging has progressed with the development of spectral-domain optical coherence tomography (SD-OCT), questions about the cause of ERM have been getting answered [4, 5]. PVD is a normal aging process which results from the liquefaction of vitreous [6]. While most of eyes show gradual PVD progress throughout the retina, some eyes show asymmetric PVD from focal adhesion of vitreous to the retinal surface [7]. This abnormal adhesion or traction of the vitreous cortex on the inner retinal surface often results in the vitreoretinal interface disorder, such as ERM, representing intraocular fibrosis [8, 9].

Sphingosine-1-phosphate (S1P) is a known mediator of fibrotic changes induced by the mechanosensitive response in several cell types [10–12]. S1P has been known as a pleiotropic growth factor in the retina that regulates proliferation, migration, differentiation, survival, and protein synthesis of various cell types [13]. Recent microarray analysis revealed that the sphingolipid metabolism was significantly affected by cellular stretching on Muller glial cell [14], which is a major cellular source of ERM [15, 16] and known to be sensitive to stretching [17].

We hypothesized that S1P may play a role in the development of human ERM that is induced by stretching of Muller glial cells during the PVD. In the present study, we evaluated S1P expression in human ERM specimens and analyzed possible clinical correlations.

## 2. Material and methods

A prospective, open-label, non-randomized interventional study was performed in patients who were scheduled for pars plana vitrectomy (PPV) for ERM removal. This study was approved by the Institutional Review Board (IRB)/Ethics Committee of KEYE EYE Center before patient recruitment (IRB approval no. P01-201910-31-004). The study protocol adhered to the tenets of the Declaration of Helsinki. All the participants signed an informed consent form after they were given a detailed explanation of the study design, associated surgical procedures for scientific purposes, and adjuvant imaging procedures.

### 2.1. Patients and clinical data acquisition

Patients who were scheduled for PPV for primary and secondary ERM removal were recruited from the Apgujung St. Mary’s Eye Center and Keye Eye Center between October 10, 2019, and December 28, 2020. The exclusion criteria were the following: (1) any pharmacological intervention in the study eye within 6 months, (2) panretinal photocoagulation in the study eye within 6 months, (3) any pharmacological intervention in the fellow eye within 3 months, (4) any history of intraocular surgery other than uncomplicated cataract surgery on the study eye, and (5) any history of ocular trauma in the study eye.

All patients underwent postoperative follow-up for 6 months. Comprehensive ocular examinations, including best-corrected visual acuity (BCVA) assessments, intraocular pressure (IOP) examinations, slit-lamp examinations, color fundus photography, and SD-OCT (Heidelberg Engineering, Heidelberg, Germany), were performed before PPV. Best-corrected visual acuity was measured using the decimal system and then converted to the logarithm of minimal angle of resolution (logMAR) units for the statistical analysis. Demographic findings, such as age, sex, and the presence proliferative diabetic retinopathy (PDR), retinal breaks, rhegmatogenous retinal detachment (RRD), and intraocular inflammation were recorded.

Various OCT findings were collected, such as central subfoveal thickness (CST), attenuation of the ellipsoid zone (EZ), and the presence of intraretinal cysts [18], lamellar holes with epiretinal proliferation (LHEP) [19], pseudoholes, and paravascular inner retinal defects, as previously described [20]. Two masked investigators (S.J., J.B.) interpreted the OCT images. When there was disagreement, the third investigator (S.K.) was consulted for the final decision.

### 2.2. Immunohistochemistry of ERM specimens

Epiretinal membrane samples were immediately fixed in a 4% (v/v) paraformaldehyde solution and washed three times with 0.1 M phosphate-buffered saline (PBS; pH 7.4). After permeabilization in 0.5% (v/v) Triton X-100 for 30 min and blocking in 10% normal goat serum for 2 h, ERM samples were incubated with rabbit anti-S1P antibody (Abcam, Cambridge, UK) at 4°C overnight and washed three times with 0.1 M PBS. Each ERM sample was then incubated with anti-rabbit antibody conjugated with rhodamine (Santa Cruz Biotechnology, Santa Cruz, CA, USA) at room temperature for 2 h and washed three times. All staining procedures were performed with ERM specimens that were placed in 2.0-ml microcentrifuge tubes as previously described [16]. After staining, the ERM samples were transferred to glass slides and mounted with ProLong Gold Antifade Mountant that contained 4′,6-diamidine-2′-phenylindole dihydrochloride‎ (DAPI; Invitrogen, Carlsbad, CA, USA). Mouse immunoglobulin G isotypes that matched those of the primary antibodies (Sigma-Aldrich, St. Louis, MO, USA) were used as negative controls. Images were taken with a microscope (EVOS M5000; Invitrogen).

To quantify cells in the ERM samples, three random images were taken at 200× final magnification, and the average number from three images was calculated and used for further analyses. The total number of cells per hyperfield (HF) was measured by detecting DAPI-positive cells in each image. The number of S1P-positive cells was measured by detecting FITC-positive cells in each image. Finally, the percentage of S1P-positive cells relative to total cells in each hyperfield was calculated. All measurements were performed by two investigators (K.S.K and J.B.) who were blinded to the clinical data and OCT findings, and the average number from each investigator was used for further analysis.

### 2.3. Human Muller glial cell culture

Spontaneously immortalized human Muller glial cells (MIO-M1) were purchased from UCL Business PLC (London, UK) and cultured in Dulbecco’s Modified Eagle Medium that contained high glucose and stable glutamine, supplemented with 10% fetal bovine serum (FBS) [21]. To assess the effect of S1P on human Muller glial cells, the cells were seeded at a density of 1 × 10^5^ cells/ml in six-well plates and treated with human S1P (10 μM; Avanti polar lipids; Alabaster, AL. USA). The S1P treatment dose was chosen by measuring the level of expression of N-cadherin according to incremental doses from 0 to 20 μM (S1 Fig). N-cadherin and NF-kB have been shown to be a key factor of S1P-related fibrosis in other cell types [22]. We used 10 μM S1P for this experiment because this dose exerted a maximal effect for those molecules on Muller glial cells.

### 2.4. Cell migration and invasion assays

To evaluate the effect of S1P on the migration of Muller glial cells, a scratch test was performed at 90% confluency. After 4 h of starvation, scratches were made using a 200 μl pipette tip. Images from randomly selected scratched areas were taken, and the width of the scratch lines was measured under 4× magnification. Transwell assays were performed to measure the number of cells that migrated toward different concentrations of S1P using transwell chambers with an 8 μm pore size insert (Corning Costar, Cambridge, USA). Cells were serum-starved for 4 h, and 2 × 10^5^ cells in 300 μl of 1% FBS medium for each insert were incubated for 12 h at 37°C in a 5% CO_2_ atmosphere. In the lower chamber, 500 μl of the same medium as the upper chamber was added. Following 12 incubations at 37°C, cells on the upper membrane were removed with a cotton swab. The filter was then immersed in methanol for 15 min at 22°C ± 2°C. The number of cells that migrated to the lower side of the membrane was counted using 0.25% crystal violet stain. Five fields of view were randomly selected under 4× magnification, and the mean number of migrated cells was quantified by microscopy. Additionally, the surface area was measured (in pixels) using Photoshop 5.5 software (Adobe, San Jose, CA, USA) as previously described [23]. Briefly, the surface area of migrated cells was determined using the “magic wand” tool (with adjustment of tolerance of 1 to 5, brush size 5, and 200% zoom). The selected area (in pixels) was divided by the total area, and the percentage of the selected area was taken for further statistical analysis (S2 Fig).

### 2.5. Real-time quantitative polymerase chain reaction and Western blot

Total RNA was extracted using TRIzol reagent (Invitrogen) according to the manufacturer’s protocol. The quantification of total RNA was performed using a NanoDrop spectrophotometer (Thermo Scientific, Waltham, MA, USA). Polymerase chain reaction (PCR) was performed in triplicate using SYBR Premix Ex Taq II (TaKaRa, Shiga, Japan) and specific primers (S1 File). The results are expressed as fold differences normalized to GAPDH using the ΔΔCt method.

Cell lysates were subjected to sodium dodecyl sulfate-polyacrylamide gel electrophoresis (SDS-PAGE), transferred to membranes, and incubated with anti-α-SMA antibody (ab5694, 1:200, Abcam), anti-N-cadherin antibody (ab18203, 1:1000, Abcam), anti-NF-kB antibody (ab32536, 1:1000, Abcam) and anti-GAPDH antibody (ab8245, 1:5000, Abcam). The membranes were then incubated with horseradish peroxidase-conjugated secondary antibodies, and proteins were visualized using a chemiluminescence substrate (Thermo Scientific).

### 2.6. Statistical analysis

SPSS 15.0 software for Windows (SPSS, Chicago, IL, USA) was used for the statistical analyses. Two-tailed Student’s *t*-test was used to assess differences in parameters according to binary variables. Values of *p* < 0.05 were considered statistically significant.

## 3. Results

### 3.1. Baseline demographic and ocular characteristics

Twenty-four consecutive patients with ERM who were scheduled for PPV and ERM removal were enrolled in this prospective interventional study. Baseline demographic characteristics of the enrolled patients for S1P staining are described in Table 1. Fifteen female and nine male patients, with a mean age of 65.65 ± 9.97 years, were included. Five patients (20.8%) had secondary ERM; One patient with PDR, two patients with a history of RRD, and two patients with history of intraocular inflammatory disease (Behcet’s diseases and CMV-associated anterior uveitis). The mean UCVA and BCVA of logMAR were 0.61 ± 0.33 and 0.37 ± 0.19, respectively.

**Table 1:** Clinical characteristics of enrolled patients (n = 24).

Characteristics	Data
Age, year	65.65 ± 9.97
Sex, male (%)	9 (37.5)
UCVA, LogMAR	0.61 ± 0.33
BCVA, LogMAR	0.37 ± 0.19
Secondary ERM, yes (%)	5 (20.8)
CST, μm	477.40 ± 104.67
Intraretinal cyst, yes (%)	11 (45.8)
LHEP, yes (%)	2 (8.3)
Pseudohole, yes (%)	2 (8.3)
PVA, yes (%)	6 (25.0)
EZ defect, yes (%)	9 (37.5)

BCVA, best-corrected visual acuity; CST, Central subfoveal thickness; ERM, epiretinal membrane; EZ, ellipsoid zone; HF, hyperfield; LHEP, lamellar hole with epiretinal proliferation; PVA, paravascular abnormality; UCVA, uncorrected visual acuity.

Epiretinal membrane specimens exhibited various ranges of cell densities, from 24.03 to 482.00 cells/HF (mean ± SD: 219.25 ± 105.45 cells/HPF). Patients with EZ defects presented significantly higher cellularity than patients without EZ defects (284.11 ± 85.39 vs 196.40 ± 91.63, respectively; *p* = 0.046; Table 2).

**Table 2:** Patient characteristics and their association with cellular properties in ERM (n = 24).

Characteristics	Total cells / HF		S1P+ cells / HF	
	Data	P value	Data	P value
Secondary ERM	Yes, 299.40 ± 144.26	0.054	Yes, 128.20 ± 135.61	0.002
	No, 198.16 ± 95.10		No, 9.68 ± 36.01	
EZ defect	Yes, 284.11 ± 85.39	0.046	Yes, 87.56 ± 117.79	0.036
	No, 196.40 ± 91.63		No, 2.80 ± 8.85	

ERM, epiretinal membrane; EZ, ellipsoid zone; HF, hyperfield.

**Fig 1:**
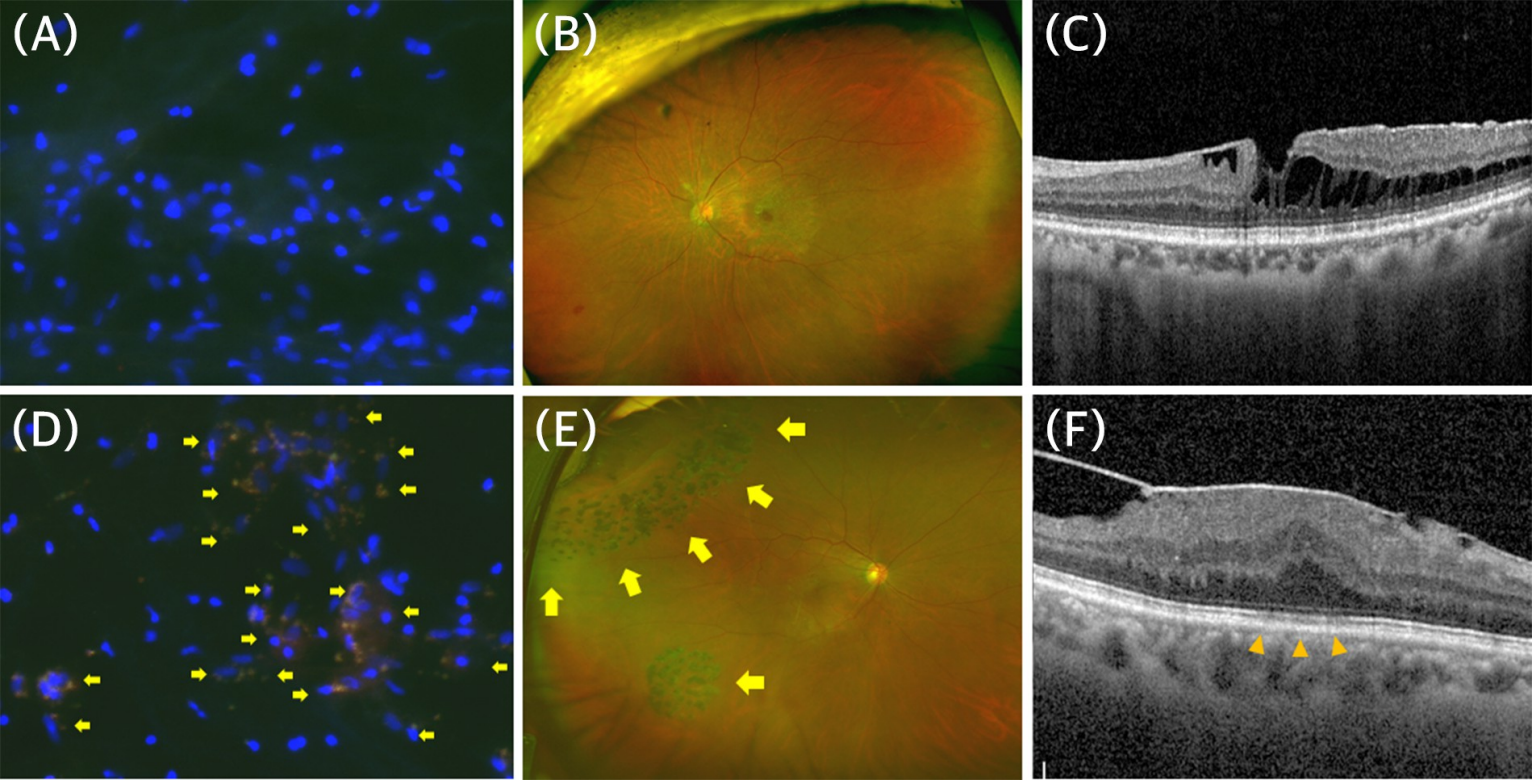
Representative images of stained epiretinal membrane (ERM) specimens and retinal images from patients with primary (A-C) and secondary (D-F) ERM. (A) Epiretinal membrane specimen from a 69-year-old female patient with primary ERM, showing negative staining for sphingosine-1-phosphate (S1P). (B) Ultrawide field retinal photograph that shows no specific underlying retinal disease. (C) Image of spectral-domain optical coherence tomography (SD-OCT) that shows ERM with partial retinoschisis but no EZ defect. (D) Epiretinal membrane specimen from a 61-year-old male patient with secondary ERM from rhegmatogenous retinal detachment (RRD), showing positive staining for S1P (arrows). (E) Ultrawide field retinal photograph that shows a retinal tear with laser scars (arrows). (F) Image of SD-OCT that shows ERM with EZ defect (arrowheads).

**Fig 2:**
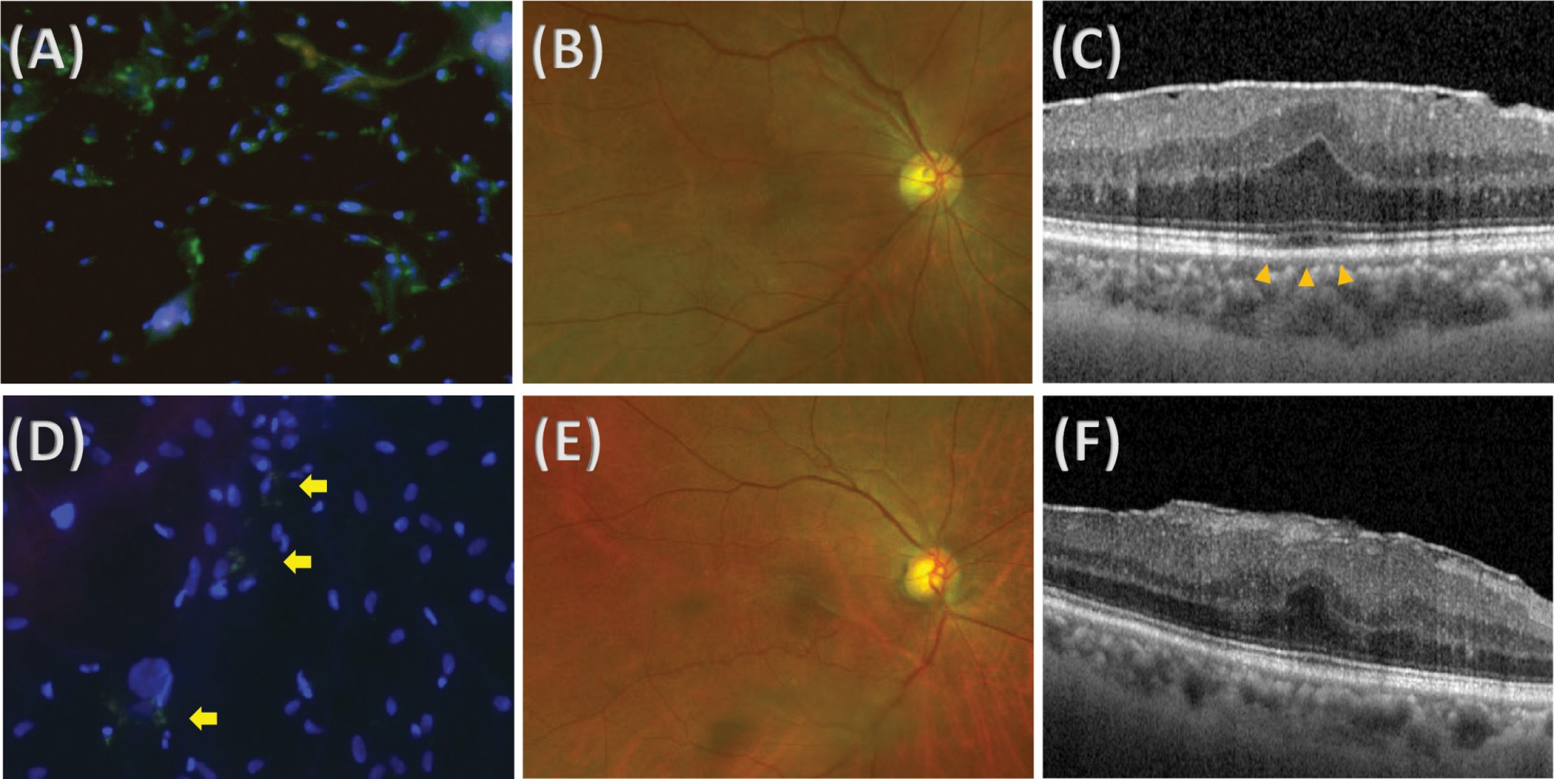
Representative images of stained epiretinal membrane (ERM) specimens and retinal images from patients with primary ERM with (A-C) or without (D-F) ellipsoid zone (EZ) defects. (A) Epiretinal membrane specimen from a 64-year-old female patient with primary ERM with EZ defect, showing higher positive staining for sphingosine-1-phosphate (S1P). (B) Ultrawide field retinal photograph that shows no specific underlying retinal disease. (C) Image of spectral-domain optical coherence tomography (SD-OCT) that shows ERM with EZ defect (arrowheads). (D) Epiretinal membrane specimen from a 73-year-old female patient with primary ERM without EZ defect, showing lower positive staining for S1P. Only a couple of cells were stained with S1P (arrows). (E) Ultrawide field retinal photography that shows no specific underlying retinal disease. (F) Image of SD-OCT that shows ERM without EZ defect.

Among 24 specimens, seven eyes (29.2%) exhibited S1P expression. S1P+ cells exhibited a heterogeneous distribution among specimens, from 0 to 334.2 cells/HF (mean ± SD: 34.38 ± 81.43 cells/HF). Patients with secondary ERM presented a significantly higher S1P+ cell density (128.20 ± 135.61 vs 9.68 ± 36.01 cells each, *p* = 0.002). Fig 1 shows representative images of stained ERM specimens from patients with primary ERM (Fig 1A–1C) and secondary ERM (Fig 1D–1F), respectively. All patients with secondary ERM presented S1P+ cells in their ERMs, whereas two of 19 patients (10.5%) with primary ERM presented S1P+ cells in their ERMs. Epiretinal membrane specimens from patients with EZ defects on SD-OCT exhibited a significantly higher S1P+ cell density (87.56 ± 117.79 vs 2.80 ± 8.85, *p* = 0.036). Fig 2 shows representative images from patients with primary ERM with S1P+ cells in the primary ERM group. Both patients who were female in their 60s had no underlying systemic disease or ocular disease. The ERM from patients with obvious EZ defects on SD-OCT exhibited a higher S1P+ cell density (Fig 2A) than the ERM from patients without obvious EZ defects (Fig 2D), whereas the overall density was similar.

### 3.2. Changes in migratory ability according to S1P

**Fig 3:**
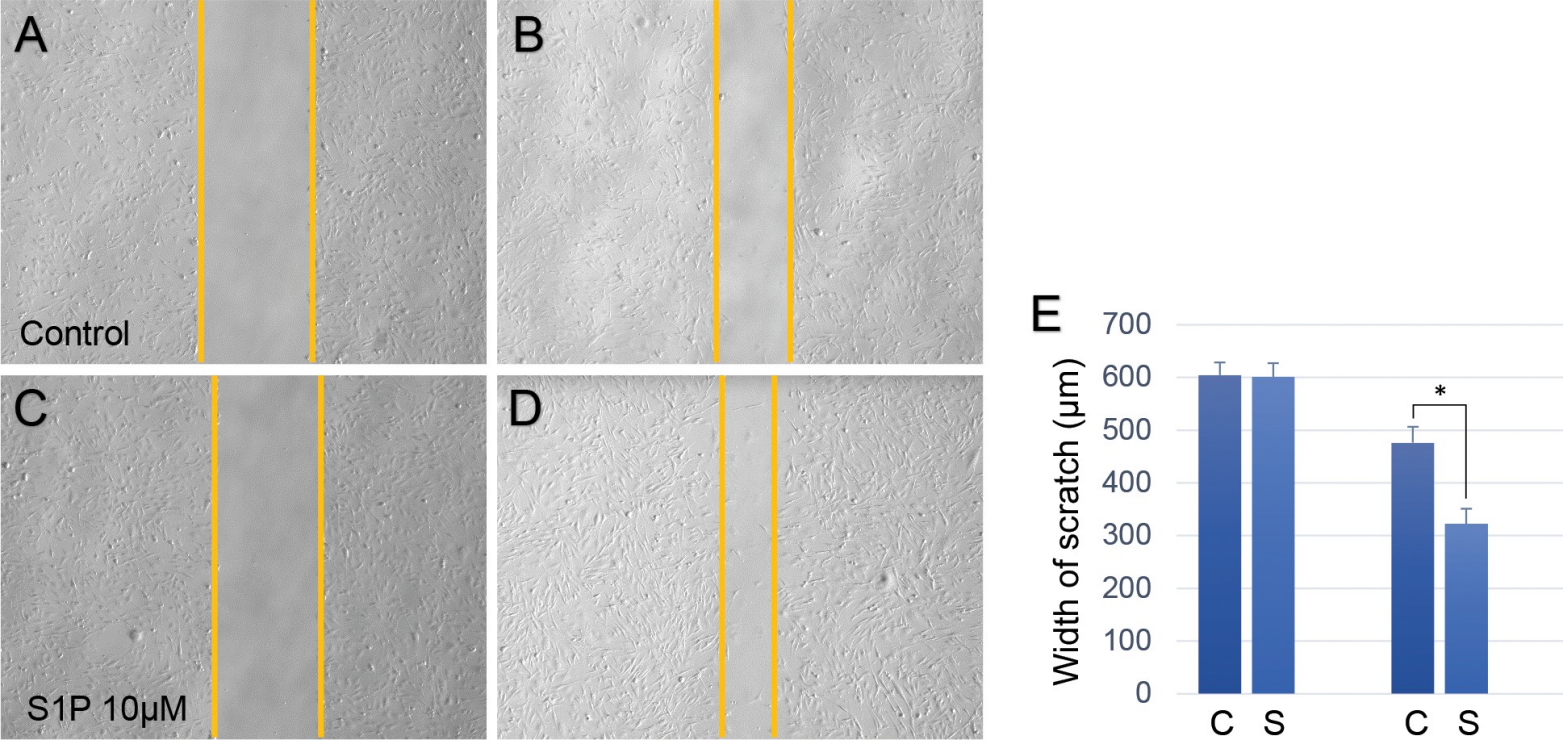
Results of the scratch test in human Muller glial cells. (A-D) Representative images of the control group at baseline (A) and 12 h later (B), sphingosine-1-phosphate (S1P) group at baseline (C) and 12 h later (D). (E) Bar graph that shows the results of the statistical analysis within groups (476.46 ± 30.03 μm for control group, 322.67 ± 28.57 μm for S1P group; p < 0.001). *p < 0.05.

A transwell test and scratch test were performed to evaluate the role of S1P in human Muller glial cells. Both tests were performed using 1% FBS medium because the higher 10% concentration of FBS has profound proliferative and migratory effects on cells (S3 and S4 Figs). Fig 3 shows the results of the scratch test. There was a significant difference in the width of scratches 12 h after treatment (control group: 476.46 ± 30.03 μm; S1P group: 322.67 ± 28.57 μm; *p* < 0.001), with no significant difference at baseline (control group: 604.80 ± 23.84 μm; S1P group: 601.13 ± 26.25 μm; *p* = 0.866).

**Fig 4:**
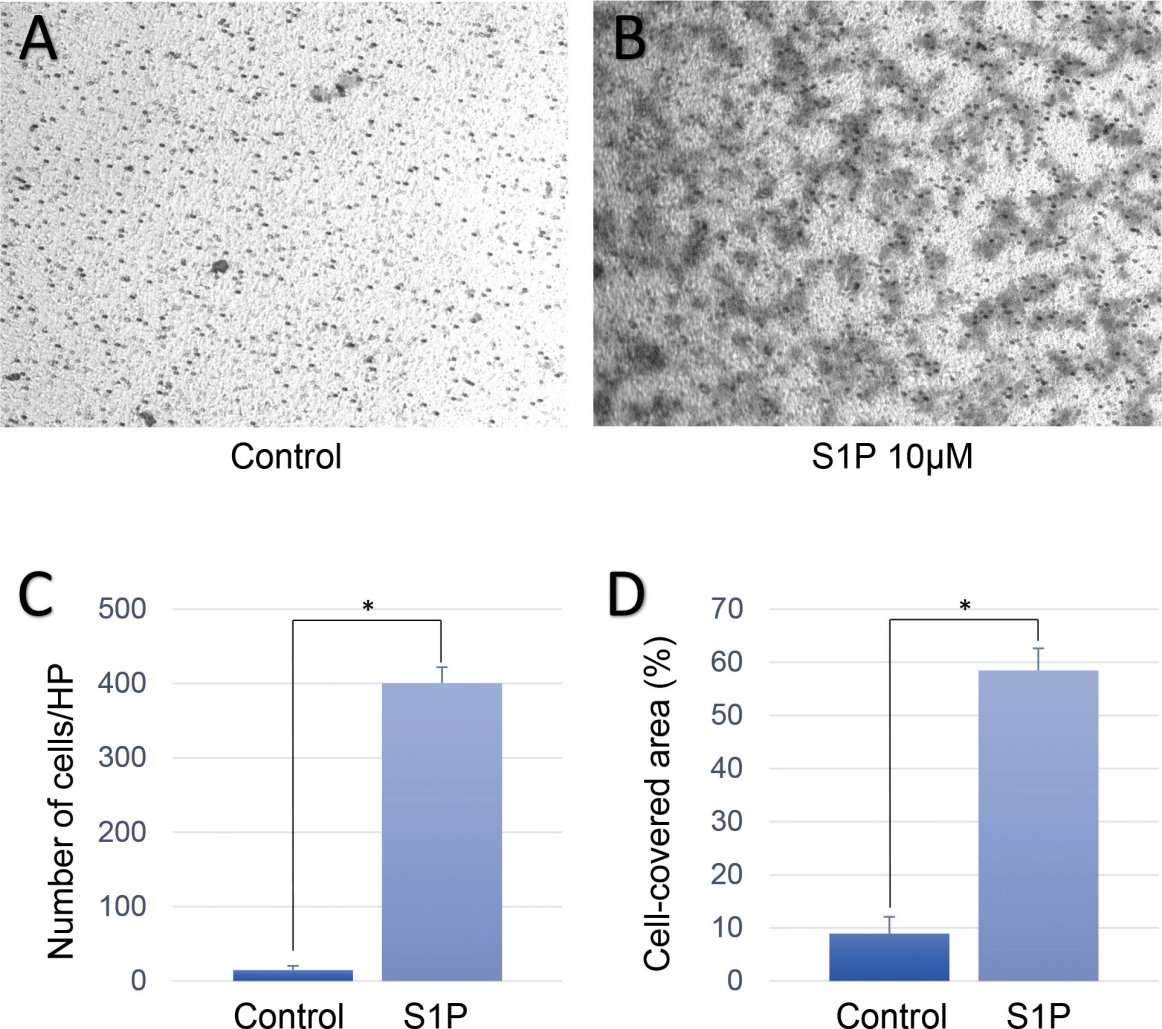
Results of the transwell test in human Muller glial cells. Representative images of the control group (A), and sphingosine-1-phosphate (S1P) group (B). (C) Bar graph that shows the results of the statistical analysis of the number of migrated cells in the different groups (15.00 ± 5.61 cells/HP for control group, 400.60 ± 21.57 cells/HP for S1P group; p < 0.001). (D) Bar graph that shows the results of the statistical analysis of the percentage of the migrated cell-covered area relative to the total area (8.94 ± 3.17 cells/HP for control group, and 58.48 ± 4.14 cells/HP for S1P group; p < 0.001). *p < 0.05.

Fig 4 shows representative images of migrated cells at the inferior surface of the transwell in each group. The transwell test showed similar results, in which Muller glial cells that were treated with S1P exhibited the more prominent migration across the filter pore than the control group (control group: 15.00 ± 5.61 cells/HP; S1P group: 400.60 ± 21.57 cells/HP; *p* < 0.001, Fig 4C). Muller glial cells that were treated with S1P covered the largest surface area than the control group (control group: 8.94 ± 3.17 cells/HP; S1P group: 58.48 ± 4.14 cells/HP; *p* < 0.001, Fig 4D).

### 3.3. Molecular change of Muller glial cells on to S1P

**Fig 5:**
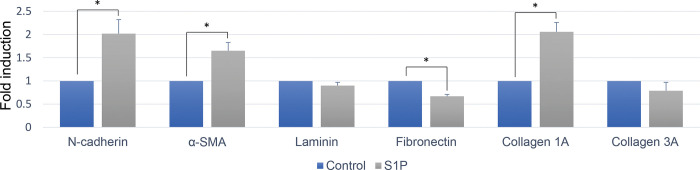
Changes in mRNA expression according to 10mM sphingosine-1-phosphate (S1P) treatment. Relative folds expression levels for indicated genes were determined by RQ-PCR with normalization to GAPDH levels (mean ± SEM, from 3 experiments, *P < 0.05).

**Fig 6:**
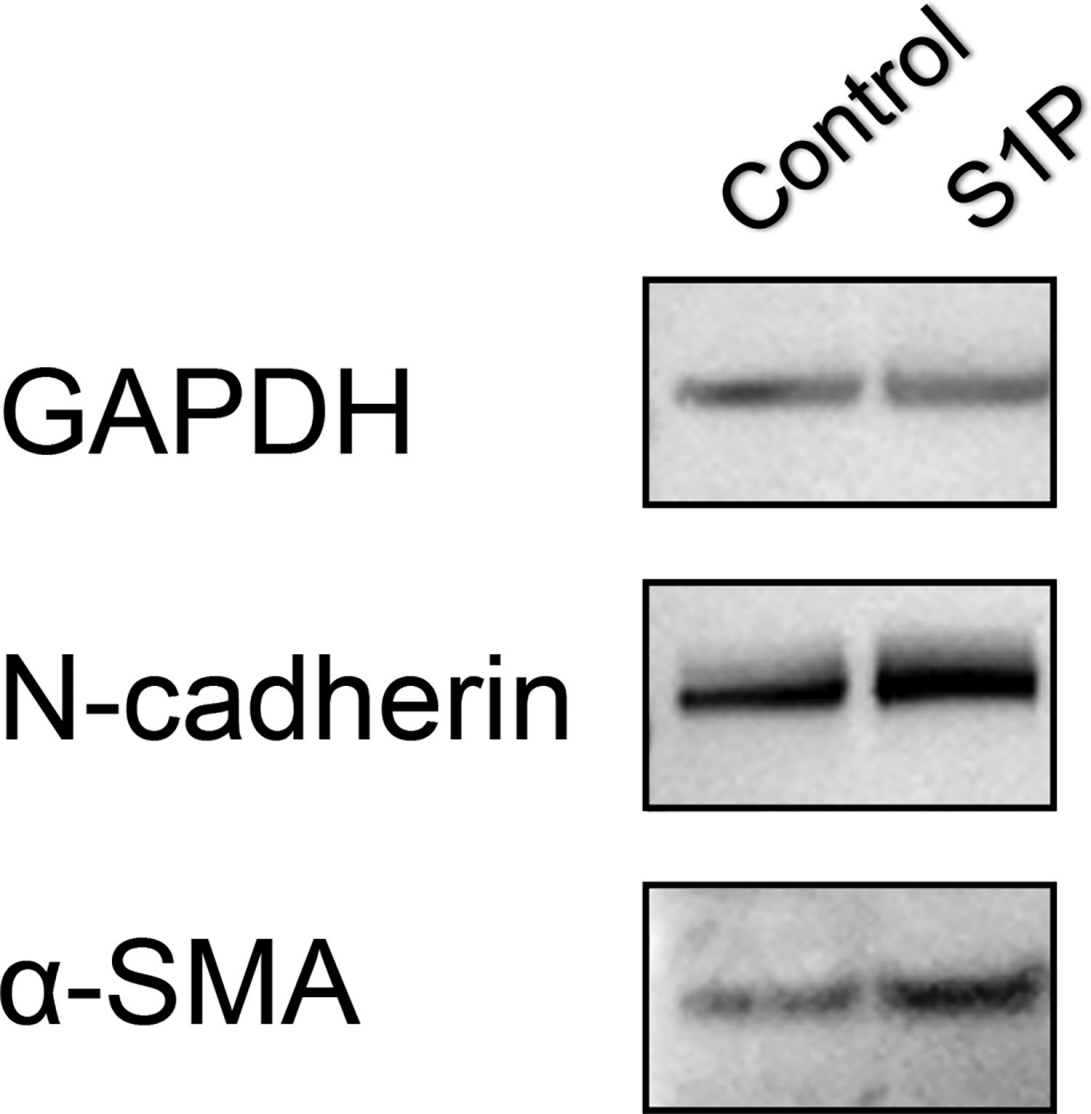
Changes in protein expression using Western blot analysis according to 10 mM sphingosine-1-phosphate (S1P) treatment. The expression of N-cadherin, and α-SMA was upregulated by S1P treatment.

To evaluate whether S1P modulates secretion of extracellular matrix proteins and results in fibrosis, changes in the mRNA expression of N-cadherin, α-SMA, laminin, fibronectin, collagen 1a, and collagen 3a were evaluated. The addition of S1P upregulated the mRNA expression of N-cadherin, α-SMA, collagen 1a and downregulated the expression of fibronectin (Fig 5). The upregulation of N-cadherin and α-SMA in Muller glial cells on to S1P was confirmed in protein level (Fig 6 and S1 Raw images).

## 4. Discussion

Although VMT that is induced by PVD has been widely accepted to initiate or aggravate ERM, the molecular pathophysiological influence of VMT on ERM has not yet been elucidated. In the present study, we found that S1P was expressed in ERM samples from both primary and secondary ERM, and S1P expression was significantly higher in secondary ERM who may experience stronger VMT. We speculated that the VMT during the PVD upregulates S1P by stretching Muller glial cells that have footplates in the internal limiting membrane (ILM). The expression of S1P was further upregulated when there was more severe traction, which may be represented as an EZ defect. EZ defects under conditions of VMT have been speculated to be a representation of photoreceptor stretching or subclinical neurosensory retinal detachment with photoreceptor disinsertion [24, 25].

The present data showed that S1P expression varied according to underlying diseases or the severity of ERM. S1P expression was higher in eyes with underlying retinal diseases, regardless of whether the disease was PDR or RRD. This was particularly interesting because we previously found that Gli1 expression was significantly higher in patients with diabetic retinopathy and significantly lower in patients with RRD [16]. We speculate that S1P expression is high in both patients with RRD and PDR because both pathologies involve severe mechanical traction between the ERM and underlying retina, whereas Gli1 expression was specifically found in patients with DR as the pathway was associated with chemical events, such as hypoxia [26].

For the development of ERM, there must be an initial escape of Muller glial cells from their original location out of the ILM, followed by migration, ECM production, and finally contraction of the ERM, which can ultimately cause metamorphopsia. In the present study, Muller glial cells exhibited significantly higher migration efficiency through small pores of the transwell insert when they were in S1P-enriched conditions, which might indicate the escape of Muller glial cells out of the ILM. Additionally, S1P-treated Muller glial cells exhibited significantly higher migratory ability in the scratch test, which may mitigate the migration of Muller glial cells over the ILM, suggesting that S1P is a component of the molecular mechanism of stepwise ERM formation that is induced by PVD. S1P increased the expression of N-cadherin, suggesting that S1P induced the EMT in Muller glial cells. Moreover, S1P increased the expression of the mature myofibroblast marker α-SMA, suggesting a transition to a more proliferative and contractile nature of Muller glial cells. In the present study, we found that the S1P is a potential causative molecule for the ERM formation in patients with strong VMT during the PVD process. Molecular pathophysiology of the initiation and aggravation of ERM can vary substantially according to underlying retinal conditions. It is important to understand the detailed molecular mechanisms to develop effective medical treatments for ERM. Our study has a limitation that we did not measure the functional effect of ERM using microperimetry or an M-chart. Further studies in a large cohort are warranted to evaluate the possible mechanisms involved in the formation of ERM according to underlying retinal conditions.

## Supporting information

S1 Fig Fold expression relative to GAPDH, detected by quantitative polymerase chain reaction of N-cadherin (A) and Western blot of NF-kB (B) according to various S1P doses.
(TIF)

S2 Fig Measurement of surface area using Photoshop 5.5 software (Adobe, San Jose, CA, USA). Areas were selected using the “magic wand” tool (black dotted lines).
(TIF)

S3 Fig Results of the scratch test in human Muller glial cells using 10% fetal bovine serum (FBS). Representative images of the control group at baseline (A) and 12 h later (B), and S1P group at baseline (C) and 12 h later (D).
(TIF)

S4 Fig Results of the transwell test in human Muller glial cells using 10% fetal bovine serum (FBS). Representative images of the control group (A) and S1P group (B).
(TIF)

S1 Raw images Full length of blots of GAPDH from Fig 6(A), N-cadherin from Fig 6(B), α-SMA from Fig 6(C), GAPDH from S2D Fig, and NF-kB from S2E Fig. (PDF)

S1 File (DOCX)
