# Supplementary figures and images for: Sphingosine-1-phosphate expression in human epiretinal membranes
Source: PLoS One. 2022 Aug 31;17(8):e0273674. doi: 10.1371/journal.pone.0273674 (PMC9432740; doi:10.1371/journal.pone.0273674)

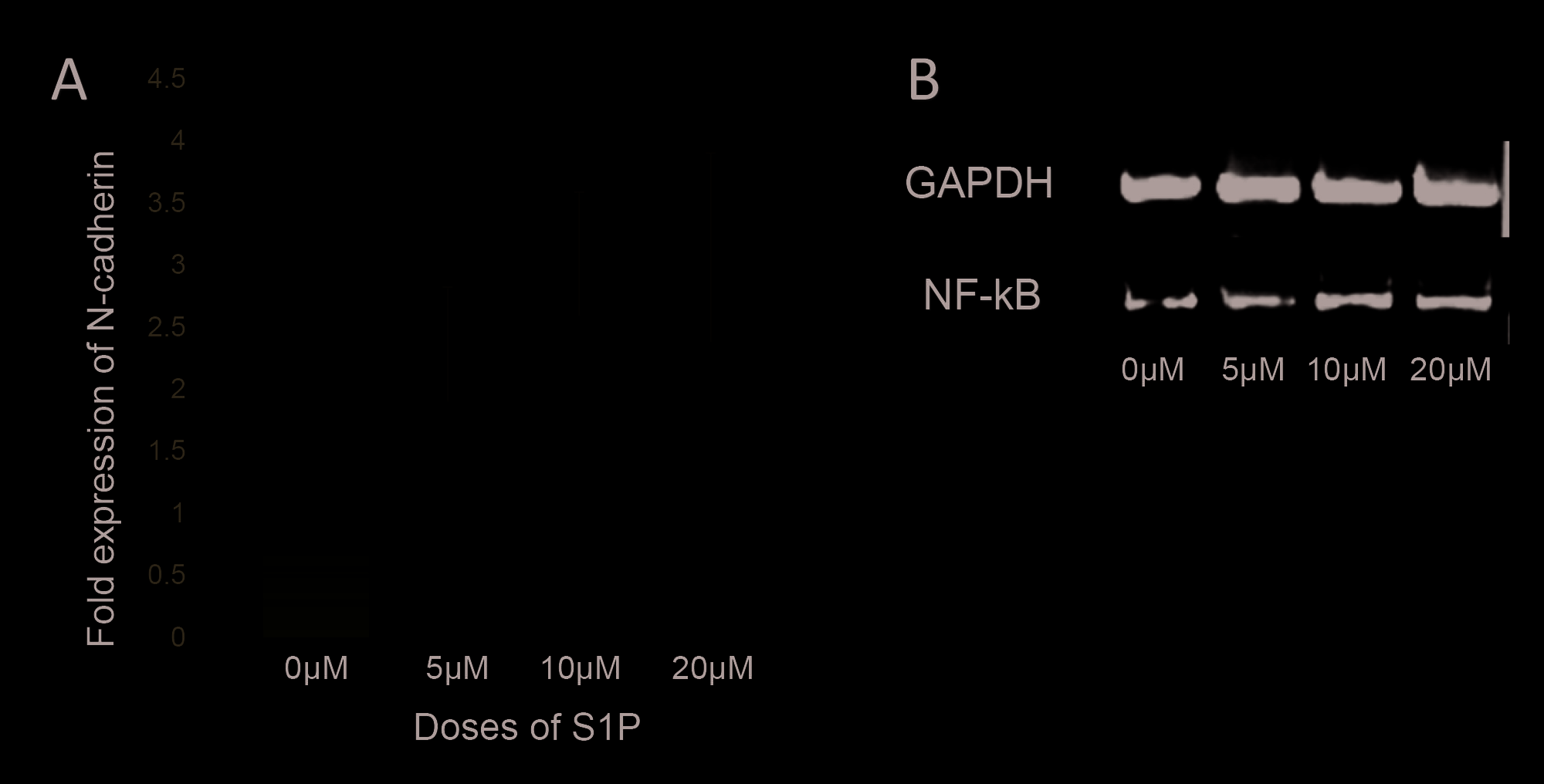

Supplement: S1 Fig — Fold expression relative to GAPDH, detected by quantitative polymerase chain reaction of N-cadherin (A) and Western blot of NF-kB (B) according to various S1P doses. (TIF) [file pone.0273674.s001.tif]

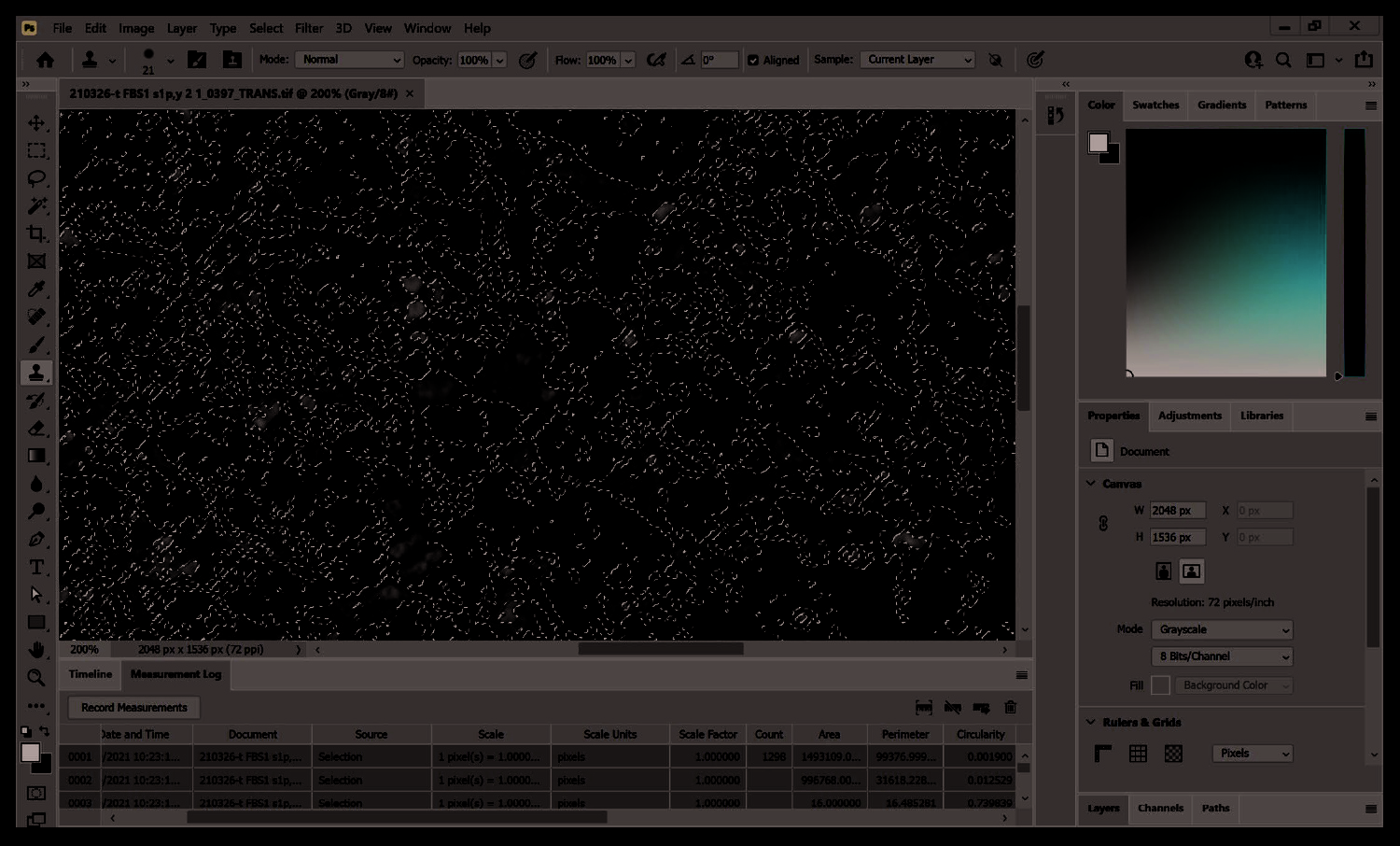

Supplement: S2 Fig — Areas were selected using the “magic wand” tool (black dotted lines). (TIF) [file pone.0273674.s002.tif]

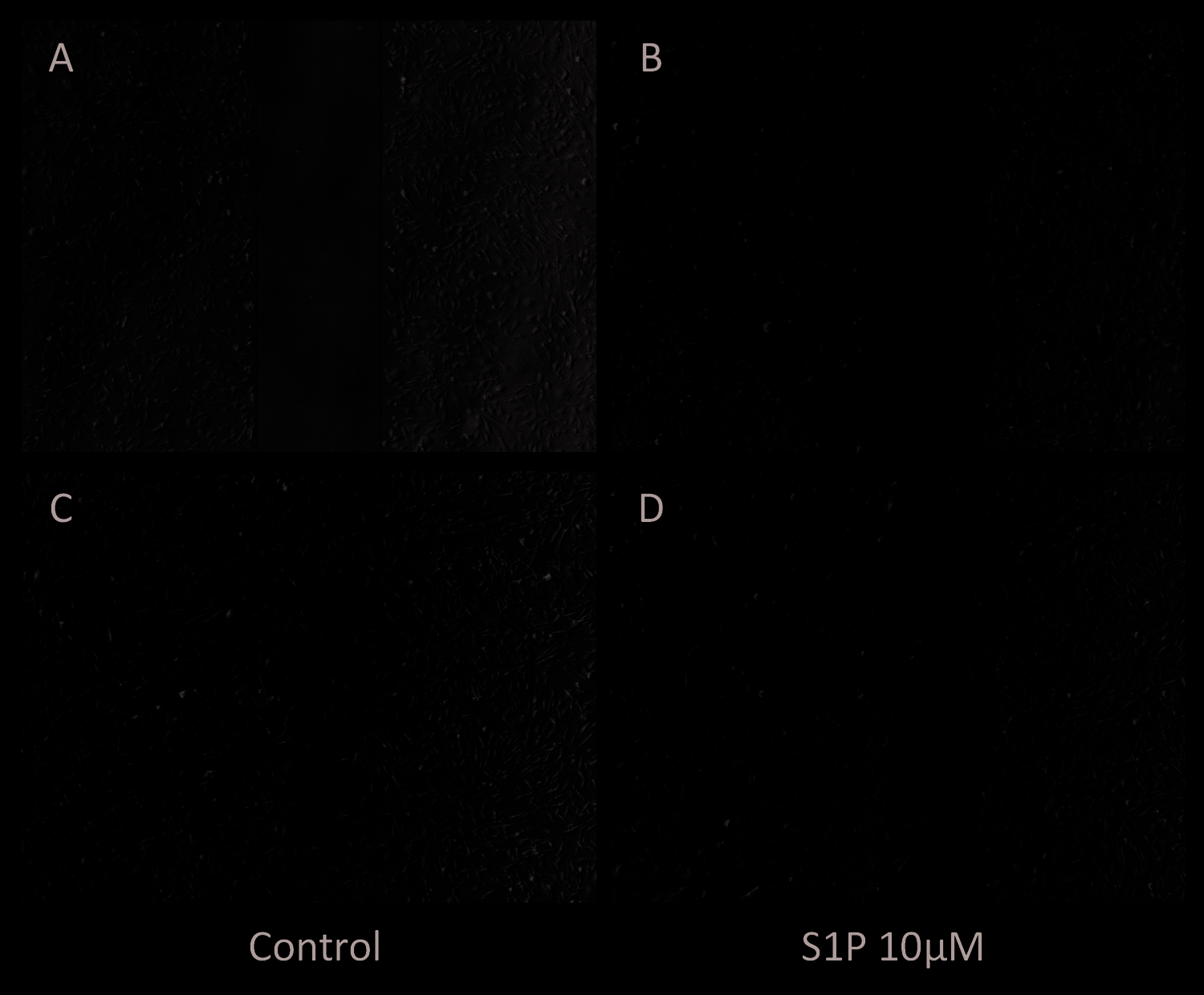

Supplement: S3 Fig — Representative images of the control group at baseline (A) and 12 h later (B), and S1P group at baseline (C) and 12 h later (D). (TIF) [file pone.0273674.s003.tif]

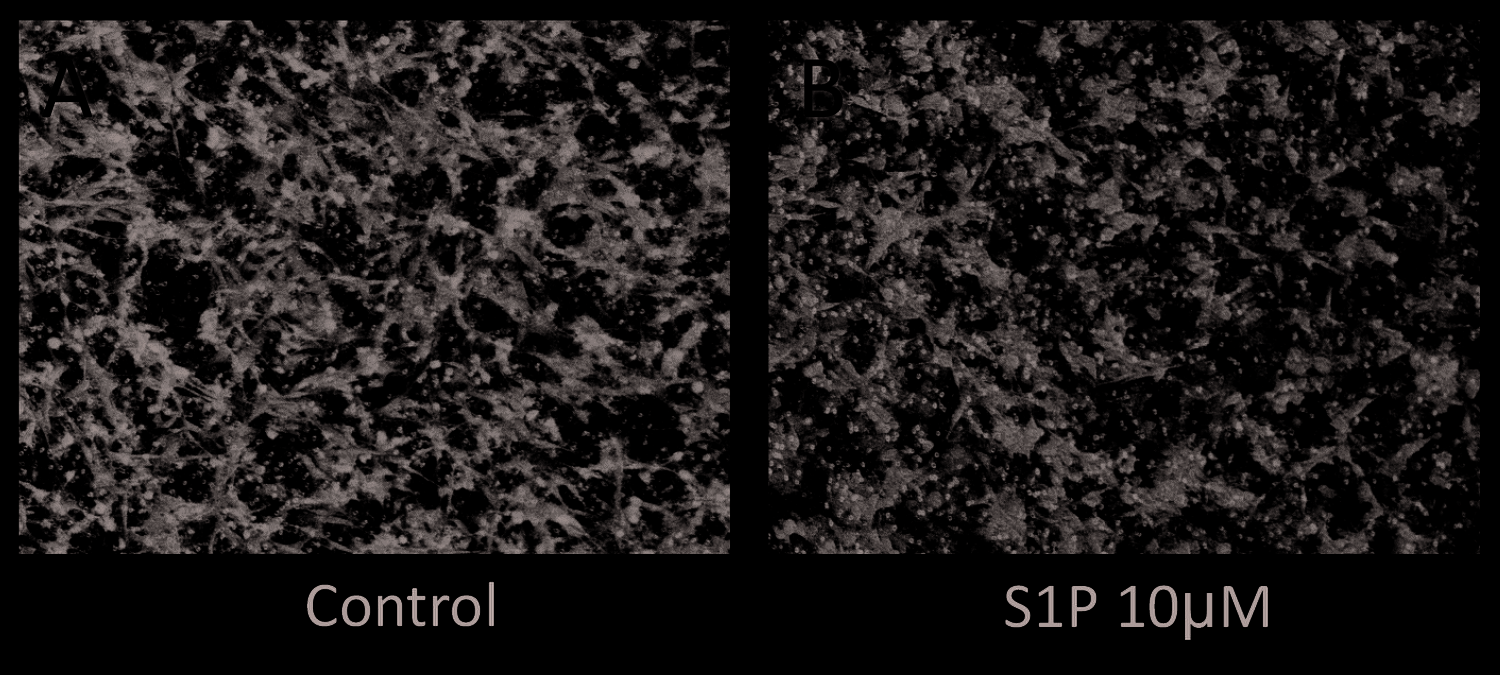

Supplement: S4 Fig — Representative images of the control group (A) and S1P group (B). (TIF) [file pone.0273674.s004.tif]
