# Supplementary material for: Sphingosine-1-phosphate expression in human epiretinal membranes
Source: PLoS One. 2022 Aug 31;17(8):e0273674. doi: 10.1371/journal.pone.0273674 (PMC9432740; doi:10.1371/journal.pone.0273674)

Figure 6

Sample:
human Muller glial cells (MIO-M1)

Method: iBright Imaging system

GAPDH (ab8245) α-SMA (ab5694) N-cadherin (ab18203)

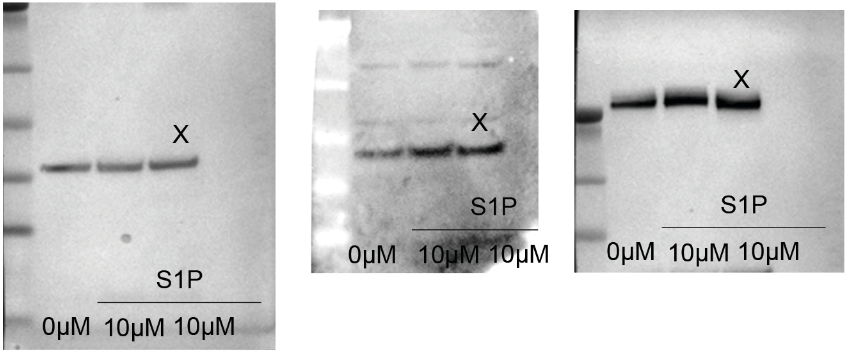

Supplemental Figure 1

Sample:
human Muller glial cells (MIO-M1)

Method: iBright Imaging system

GAPDH (ab8245) NF-kB (ab32536)

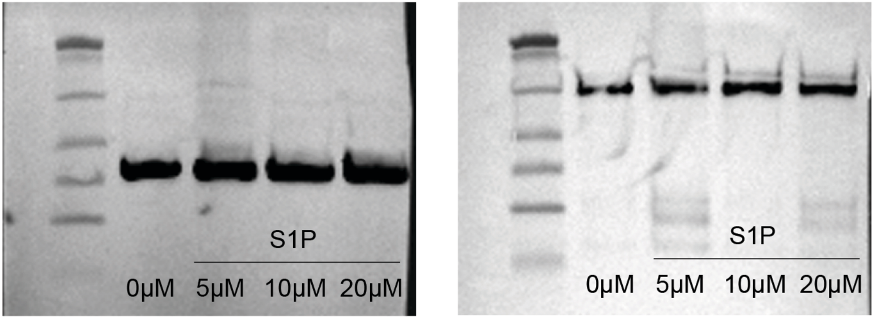

Supplement: S1 Raw images — (PDF) [file pone.0273674.s005.pdf]
