# Supplementary material for: Sphingosine-1-phosphate expression in human epiretinal membranes
Source: PLoS One. 2022 Aug 31;17(8):e0273674. doi: 10.1371/journal.pone.0273674 (PMC9432740; doi:10.1371/journal.pone.0273674)
Supplement: S1 File — (DOCX) [file pone.0273674.s006.docx]

**Supplemental Information**

Supplemental Figure S1. Measurement of surface area using Photoshop 5.5 software (Adobe, San Jose, CA, USA). Areas were selected using the “magic wand” tool (black dotted lines).

Supplemental Figure S2. Fold expression relative to GAPDH, detected by quantitative polymerase chain reaction of N-cadherin (A) and Western blot of NF-kB (B) according to various S1P doses.

Supplemental Figure S3. Results of the scratch test in human Muller glial cells using 10% fetal bovine serum (FBS). Representative images of the control group at baseline (A) and 12 h later (B), and S1P group at baseline (C) and 12 h later (D).

Supplemental Figure S4. Results of the transwell test in human Muller glial cells using 10% fetal bovine serum (FBS). Representative images of the control group (A) and S1P group (B).

Supplemental Figure S5. Full length of blots of GAPDH from Figure 6 (A), N-cadherin from Figure 6 (B), α-SMA from Figure 6 (C), GAPDH from supplemental figure 2 (D), and NF-kB from supplemental figure 2 (E).

**Primer sequences used in the present study.**

GAPDH-F: CCCCACCACACTGAATCTCC

GAPDH-R: GGTACTTTATTGATGGTACATGACAAG

N-cadherin-F: CACTGCTCAGGACCCAGAT

N-cadherin-R: TAAGCCGAGTGATGGTCC

α-SMA-F: CCGACCGAATGCAGAAGGA

α-SMA-R: ACAGAGTATTTGCGCTCCGAA

Laminin-F: GAACCCGCAGTGTCGAATCT

Laminin-R: GGGGAGTTAGCTGCCTTCA

Fibronectin-F: CAGTGGGAGACCTCGAGAAG

Fibronectin-R: TCCCTCGGAACATCAGAAAC

Collagen 1A-F: GTCACCCACCGACCAAGAAACC

Collagen 1A-R: AAGTCCAGGCTGTCCAGGGATG

Collagen 3A-F: AGGTCCTGCGGGTAACACT

Collagen 3A-R: ACTTTCACCCTTGACACCCTG
